# Treatment of Open-Angle Glaucoma and Ocular Hypertension with Preservative-Free Tafluprost/Timolol Fixed-Dose Combination Therapy: Results from the VISIONARY Study Population in Spain

**DOI:** 10.1089/jop.2021.0099

**Published:** 2022-04-06

**Authors:** Jose J. Garcia-Medina, Javier Benitez-del-Castillo, Iñaki Rodríguez-Agirretxe, Fernando Lopez-Lopez, Antonio Moreno-Valladares

**Affiliations:** ^1^ Department of Ophthalmology, Hospital General Universitario Morales Meseguer, Murcia, Spain.; ^2^ Department of Ophthalmology, Hospital Universitario de Jerez, Jerez de la Frontera Cadiz, Spain.; ^3^ Department of Ophthalmology, Hospital Universitario Donostia, San Sebastian Guipuzcoa, Spain.; ^4^ Instituto Oftalmológico Gomez-Ulla, Santiago de Compostela Galicia, Spain.; ^5^ Department of Ophthalmology, Complejo Hospitalario Universitario de Albacete, Albacete, Spain.

**Keywords:** open-angle glaucoma, ocular hypertension, tafluprost, timolol, fixed-dose combination, preservative-free topical medication

## Abstract

**Purpose:**

Data are presented from ophthalmology clinics in Spain participating in the VISIONARY study, examining the effectiveness, tolerability, and safety of the preservative-free tafluprost (0.0015%) and timolol (0.5%) fixed-dose combination (PF tafluprost/timolol FC) in the treatment of OAG and OHT.

**Methods:**

An observational, multicenter prospective study examined treatment outcomes following a switch to PF tafluprost/timolol FC in adult OAG/OHT patients demonstrating insufficient response to beta-blocker or prostaglandin analog (PGA) monotherapy. Primary end point was mean change in intraocular pressure (IOP) from baseline at month 6. Changes in the severity of ocular signs and symptoms were also assessed.

**Results:**

Overall, 92 patients (51.1% female) were included. Mean (standard deviation) age was 68.3 (12.1) years. Mean IOP was reduced from 21.9 mmHg at baseline to 16.7 mmHg at month 6 (22.3% decrease; *P* < 0.0001). Significant IOP reductions were observed at weeks 4 and 12 (*P* < 0.0001). Baseline PGA and beta-blocker users demonstrated mean month 6 IOP reductions of 5.5 mmHg (23.5%; *P* < 0.001) and 3.5 mmHg (14.6%; *P* = 0.029), respectively. Severity of conjunctival hyperemia, dry eye, irritation, itching, foreign body sensation, and eye pain was significantly reduced. Three treatment-related adverse events were reported, all were nonserious and mild/moderate in severity.

**Conclusion:**

In real-world clinical practice, PF tafluprost/timolol FC treatment provided significant IOP reductions over 6 months and was well tolerated among OAG/OHT patients showing poor response to PGA or beta-blocker monotherapy. IOP-lowering efficacy and improvements in ocular signs and symptoms were evident from week 4 and maintained over the 6-month study period. Trial Registration: European Union electronic Register of Post-Authorisation Studies (EU PAS) register number EUPAS22204.

## Introduction

Glaucoma is a leading cause of blindness worldwide. The combined global prevalence of primary open-angle glaucoma (POAG) and primary angle-closure glaucoma has been estimated to be ∼3.54% (64.3 million) among people aged 40–80 years, with the number of people living with the disease being projected to reach 111.8 million by the year 2040.^1^ Data concerning the prevalence of glaucoma in Spain are lacking. However, a study of people aged between 40 and 79 years living in the province of Segovia revealed that POAG was present in ∼2.1% of this population, which is in line with estimates across Europe.^2,3^

Elevated intraocular pressure (IOP) is the most important modifiable risk factor for development and progression of open-angle glaucoma (OAG), and available evidence suggests that reduction of IOP may slow visual field loss.^4–11^ Recommended treatments for OAG include topical prostaglandin analog (PGA), beta-blocker, carbonic anhydrase inhibitor, or α_2_-adrenergic agonist monotherapy.^12^ Initially, monotherapy is advised and treatment may be intensified through the addition of one or more IOP-lowering medications in cases of uncontrolled IOP.^12^

Studies indicate that increasing complexity of treatment regimen negatively affects compliance across a range of chronic conditions, including glaucoma.^12–14^ Fixed-dose combination (FC) treatment is therefore preferred to the instillation of multiple separate therapies—to simplify instillation and aid long-term adherence with therapy.^12,15–17^

PGA and beta-blocker FC therapies are commonly prescribed glaucoma medications.^12,15–21^ Treatment must balance efficacy with tolerability; long-term exposure to preservative-containing glaucoma medications, particularly the commonly used benzalkonium chloride (BAK), is associated with ocular surface toxicities that can lead to development of ocular surface disease and increased risk of future filtration surgery failure.^22–27^ Preservative-free (PF) FC therapies reduce daily instillations and exposure to preservative agents.^5,12,15,17–19^

The combination of PF tafluprost (0.0015%) and timolol (0.5%) in an FC formulation (PF tafluprost/timolol FC) has demonstrated efficacy alongside a good tolerability profile in randomized controlled trials (RCTs).^18,28–33^ More recently, the PF tafluprost/timolol FC has shown improvements regarding IOP-lowering efficacy and tolerability in observational studies that reflect real-world clinical practice.^34–36^

Although RCTs remain the gold standard for regulatory assessment of efficacy and safety, strict inclusion and exclusion criteria mean that many aspects of routine clinical practice cannot be effectively examined in this setting.^37^ Observational studies provide opportunities to explore important aspects of OAG and ocular hypertension (OHT) treatment that are pertinent to real-life clinical situations. In practice, glaucoma treatment switches occur without the washout period usually mandated in RCT protocols, making real-world studies more relevant and helpful in understanding how patients may tolerate and respond to changes in medication. Observational studies also provide important pharmacovigilance information and data. For these reasons, real-world evidence is becoming increasingly welcomed by regulatory bodies.^37–39^

This article reports country level data from Spain concerning the VISIONARY study; a multicenter, European observational study that evaluated the IOP-lowering effectiveness and tolerability of the PF tafluprost/timolol FC over a 6-month period in people with OAG and OHT.^34^ Participants were previously treated with either a topical PGA or a topical beta-blocker monotherapy; the most frequent first-line treatments used in practice, as suggested by the European Glaucoma Society.^12^

Country level data reported from the United Kingdom, Hungary, and Russia showed that the baseline IOP varied among patients participating in the VISIONARY study from different geographical locations.^34,40–42^ The mean IOP at baseline was 21.5, 20.8, 22.0, and 23.8 mmHg for patients in the full VISIONARY population, Hungary, the United Kingdom, and Russia, respectively, with the variation observed reflecting the real-world clinical setting in which the study was conducted and the patients deemed appropriate for treatment escalation by ophthalmologists in each country.^34,40–42^ Consequently, while statistically significant IOP reductions were reported in each of the published analyses, the mean IOP reduction achieved in each cohort varied slightly because pretreatment pressure tends to be predictive of IOP-lowering outcomes with topical therapies.^30,34,40–42^ At month 6, respective IOP reductions from baseline reported for the full VISIONARY population, Hungary, the United Kingdom, and Russia were 5.7 mmHg (24.9%), 5.0 mmHg (22.6%), 5.8 mmHg (24.9%), and 7.1 mmHg (28.1%).^34,40–42^ The country level differences observed in these Northern and Eastern European populations may be due to patient-related aspects, including race, lifestyle, and compliance, or they may be a result of variations in clinical practice or approaches to the management of glaucoma/OHT.

The current study, examining treatment outcomes achieved with PF tafluprost/timolol FC therapy in Spanish ophthalmology clinics, is the first to report results from a Southern European VISIONARY population and allows these data to be explored alongside those reported for the full VISIONARY study dataset and also in the context of the country level data published so far from the same study. This analysis of the Spanish VISIONARY dataset represents the largest and longest real-world study of PF tafluprost/timolol FC therapy in Spain. These data may provide a greater understanding of local practice regarding glaucoma treatment in Spain and the levels of IOP-lowering efficacy and tolerability that ophthalmologists and their patients should expect with PF tafluprost/timolol FC therapy.

## Methods

### Study design and visit schedule

Analyses were performed using data from OAG and OHT patients living in Spain who had been included in the VISIONARY study, comprising a 6-month, European, observational, multicenter prospective study that was conducted in a real-world clinical practice setting.^34^ The study complied with the principles of the Declaration of Helsinki. All patients included were required to provide prior written informed consent, and the study protocol was approved by the institutional review board (IRB) or independent ethics committee (IEC) at each participating center/institution (details of Spanish institutions are listed in the Acknowledgments section).

Data were prospectively collected during routine visits, between April 10, 2017 and January 9, 2019, at 10 ophthalmology clinics in Spain. Participants were required to attend the baseline and month 6 study visits. However, attendance at week 4 and 12 interim visits was optional.

### Patient population and study treatment

Male/female adults (aged ≥18 years) with a diagnosis of OAG or OHT who were currently insufficiently treated with, or showed poor tolerance to, topical PGA or a beta-blocker monotherapy were included. Baseline measures were recorded within the 7 days before initiation of PF tafluprost/timolol FC. Variables were recorded for each eye separately at all visits, with the eye having the higher baseline IOP value being selected as the study eye. If both eyes had the same IOP, then data from the right eye was considered. The investigator was able to indicate more than 1 reason for switching to PF tafluprost/timolol FC treatment, and options comprised insufficient IOP control or progression of glaucoma on the current monotherapy, conversion of OHT to OAG, poor local tolerance of the current therapy, insufficient adherence, or other reasons.

Participants were treatment naive for PF tafluprost/timolol FC therapy, had not undergone ophthalmic surgery (within 6 months), and were not pregnant or breast feeding. Those with any contraindication against tafluprost or timolol treatment were not allowed to enter the study. Participants treated their affected eye(s) with PF tafluprost/timolol FC (1 drop, administered once per day), instilled either in the morning or in the evening.

### Efficacy, tolerability, and safety evaluations

Primary end point was absolute mean IOP change (mmHg) from baseline at month 6, following initiation of PF tafluprost/timolol FC treatment. IOP was measured at baseline and at each subsequent study visit using Goldmann applanation tonometry.^12^

Secondary end points comprised mean IOP change from baseline at interim visits (week 4 and 12), responder rate, and change in severity of ocular signs and symptoms during the study period. Responder rate was defined as the proportion (%) of patients showing an IOP change from baseline of ≥20% at week 12. Responder rate analysis also examined the proportion of patients achieving a change in mean IOP from baseline of ≥20%, ≥25%, ≥30%, and ≥35% at each visit. IOP subanalysis assessed the mean IOP change from baseline according to diagnostic group (for subgroups that included >10 patients), prior monotherapy type (PGA or beta-blocker), reported reasons for switching to PF tafluprost/timolol FC treatment, the presence or absence of dry eye symptoms, and treatment instillation time (morning or evening).

A 4-grade scale (none, mild, moderate, and severe) was used to evaluate the change in severity of conjunctival hyperemia and ocular symptoms (comprising irritation, itching, dry eye feeling, foreign body sensation, and eye pain) from baseline at month 6, with optional collection of these data where patients attended interim study visits. Other parameters were optional assessments at all study visits and included corneal fluorescein staining (CFS; Oxford Grade Scale; grades 0 to V), Schirmer's test, and tear break up time (TBUT).^43^

Investigators evaluated treatment effectiveness, clinical signs, and patient compliance with PF tafluprost/timolol FC therapy, versus prior PGA or beta-blocker monotherapy, using a 3-grade scale (better than prior medication, same as prior medication, worse than prior medication). Patients assessed tolerability with PF tafluprost/timolol FC treatment using a 4-grade scale (very good, good, satisfactory, poor).

Best corrected visual acuity (BCVA) data were documented at all study visits. Visual acuity data were recorded in decimal, logMAR, or fraction (foot or meters) scales (according to the standard practice and the study center) and were converted to the decimal scale using conversion formulas, where required.^44^ Adverse events (AEs) and treatment-related AEs were recorded at all visits.

### Statistical analyses

ICON Plc (Dublin, Ireland) conducted statistical analyses on behalf of the VISIONARY study group. For normally distributed data, the mean and standard deviation (SD) were presented, and the paired *t*-test was used to assess statistical significance. Where data were not normally distributed, the median values and the interquartile range (IQR) were given and the Wilcoxon signed rank test was used to assess the change from baseline. A linear mixed model used IOP as the dependent variable and all time points as independent variables to investigate time-dependent IOP changes. Change from baseline concerning CFS, conjunctival hyperemia, and subjective symptoms was assessed using the Bhapkar test.^45^ The *P* value used as the cutoff for statistical significance was of <0.05.

## Results

### Study population demographics

In total, 129 patients were screened for the VISIONARY study from Spain, of which 92 met the inclusion criteria and completed the study. Table 1 shows the baseline demographics for the VISIONARY population in Spain. Mean age (SD) was 68.3 (12.1; range 39.7–89.8) years and 51.1% were female. In the majority of cases (70.7%), the right eye was the study eye, and patients were diagnosed with POAG (60.9%), OHT (26.1%), pseudoexfoliative glaucoma (9.8%), and normal tension glaucoma (2.2%). Most participants (87.0%) were treated with PGA monotherapy before baseline, while 13.0% were using beta-blocker therapy.

**Table 1.** Demographics of the Participants

Sex, *n* (%)	
Male	45 (48.9)
Female	47 (51.1)
Age (years)	
Mean ± SD	68.3 ± 12.1
Range	39.7–89.8
Diagnosis, *n* (%)	
POAG	56 (60.9)
OHT	24 (26.1)
Pseudoexfoliative glaucoma	9 (9.8)
Normal tension glaucoma	2 (2.2)
Pigmentary glaucoma	0 (0)
Other glaucoma	1 (1.1)
Study eye, *n* (%)	
Right	65 (70.7)
Left	27 (29.3)
Previous treatment, *n* (%)	
β-blocker therapy	12 (13.0)
PGA therapy	80 (87.0)
IOP at baseline, mmHg (mean ± SD)	21.9 ± 3.97
CFS score (Oxford grade scale) (mean ± SD) (*n* = 51)	0.3 ± 0.55
BCVA decimal score, median (IQR) (*n* = 85)	0.8 (0.50)
Schirmer's test, median (IQR) (*n* = 14)	14.5 (4.0)
TBUT seconds, median (IQR) (*n* = 15)	11.0 (5.0)

BCVA, best corrected visual acuity; CFS, corneal fluorescein staining; IOP, intraocular pressure; IQR, interquartile range; OHT, ocular hypertension; PGA, prostaglandin analog; POAG, primary open-angle glaucoma; SD, standard deviation; TBUT, tear break up time.

### Mean IOP change from baseline with PF tafluprost/timolol FC treatment

**FIG. 1.**
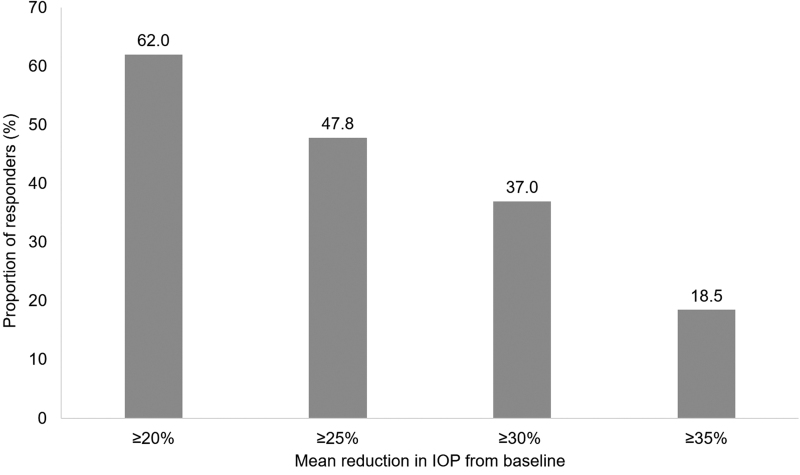
Percentage of responders according to different IOP reduction cutoff values at month 6. IOP, intraocular pressure.

The mean (SD) IOP was reduced from 21.9 (3.97) at baseline to 16.7 (3.51) mmHg at month 6, representing a decrease of 5.2 (4.04) mmHg and an overall IOP reduction of 22.3%, which was statistically significant (*P* < 0.0001) (Table 2). Mean (SD) IOP was significantly reduced from baseline at week 4 and 12, with respective IOP reductions of 5.5 (3.08) mmHg (24.3%; *P* < 0.0001) and 5.6 (3.25) mmHg (24.5%; *P* < 0.0001) demonstrated. Responder rates at month 6 based on cutoff values for reduction in mean IOP of ≥20%, ≥25%,≥30%, and ≥35% were 62.0%, 47.8%, 37.0%, and 18.5%, respectively (Fig. 1).

**Table 2.** Change in Intraocular Pressure from Baseline at Week 4, Week 12, and Month 6

Visit	*n*	Mean (SD) IOP (mm Hg)	Mean (SD) reduction in IOP from baseline (mm Hg)	Mean percentage reduction in IOP from baseline	*P* ^a^
Baseline	92	21.9 (3.97)			
Week 4	85	16.6 (3.63)	5.5 (3.08)	24.3	<0.0001
Week 12	74	16.3 (3.07)	5.6 (3.25)	24.5	<0.0001
Month 6	92	16.7 (3.51)	5.2 (4.04)	22.3	<0.0001

^a^ Significance testing using 2-sided paired *t*-test for change in mean IOP from baseline to week 4, week 12, and month 6.

Most patients were diagnosed with POAG (60.9%) or OHT (26.1%), and the mean (SD) baseline IOP in each respective group was 21.6 (3.92) and 23.0 (3.91) mmHg. Mean (SD) IOP reduction was 5.2 (3.62) and 6.3 (4.33) mmHg at month 6 among patients with POAG and OHT, respectively (*P* < 0.0001). This represented a relative change of 22.7% (POAG) and 25.8% (OHT) from baseline.

Statistical analysis was not conducted for pseudoexfoliative glaucoma and normal tension glaucoma subgroups due to low subject numbers.

For those treated with PGA monotherapy before starting PF tafluprost/timolol FC, mean (SD) IOP was 22.2 (3.78) mmHg at baseline and 16.7 (3.49) mmHg at month 6, representing a reduction from baseline of 5.5 (3.88) mmHg (23.5%) (*P* < 0.0001). Patients treated with latanoprost at baseline demonstrated mean (SD) IOP reductions of 5.6 (3.67) mmHg (24.1%; *P* < 0.0001) at Month 6. Mean (SD) IOP was 22.2 (3.84) mmHg at baseline and 16.6 (3.16) mmHg at Month 6 in the prior latanoprost subgroup. Due to low patient numbers, statistical analysis was not conducted for other PGA subgroups. For participants using beta-blocker therapy before the treatment switch, mean (SD) was reduced from 20.4 (4.98) mmHg at baseline to 16.9 (3.87) mmHg at month 6, providing a reduction of 3.5 (4.83) mmHg (14.6%) (*P* = 0.029).

The main reason for a switch to the PF tafluprost/timolol FC from PGA or beta-blocker monotherapy was insufficient IOP control (87.0%) or progression of glaucoma (19.6%), and these patients demonstrated mean (SD) IOP reductions from baseline of 5.5 (3.82) mmHg (23.6%) at month 6 (*P* < 0.0001).

Those who did not have dry eye symptoms before initiating PF tafluprost/timolol FC treatment demonstrated reductions in mean (SD) IOP of 4.9 (4.10) mmHg (21.2%) from baseline at month 6 (*P* < 0.0001). Participants with dry eye symptoms at baseline showed a reduction in mean (SD) IOP of 5.5 (3.75) mmHg (23.1%) at month 6 (*P* < 0.0001). The majority of participants (96.7%) instilled PF tafluprost/timolol FC treatment in the evening, and mean (SD) relative IOP reduction from baseline at month 6 was 5.2 (4.06) mmHg (22.3%) in this group (*P* < 0.0001). Only one patient that finished the study used morning instillation.

### Change in severity of clinical signs and subjective symptoms

Mean (SD) CFS score (Oxford Grade Scale grades 0 to V) at baseline was 0.33 (0.55). CFS score decreased at all visits during the study period, although the change was not statistically significant (*P* < 0.7668; Table 3). Median (IQR) Schirmer's test result was increased from 14.5 (4.0) at baseline to 16.0 (4.00) at month 6 (*P* = 0.003). Median (IQR) TBUT was increased from 11.0 (5.0) s at baseline to 14.0 (5.00) s at month 6 (*P* < 0.001). Median (IQR) BCVA decimal score was 0.8 (0.5) at baseline and 0.8 (0.40) at month 6.

**Table 3.** Change in Corneal Fluorescein Staining Score (Oxford Grade Scale) During the Study Period

	Mean change from baseline				
	n	Mean (SD)	n^b^	Mean (SD)	P^a^
Baseline	51	0.33 (0.55)			
Week 4 (±7 days)	57	0.23 (0.46)	45	0.02 (0.50)	0.7668
Week 12 (±7 days)	54	0.24 (0.51)	41	0.01 (0.62)	0.3233
Month 6 (±45 days)	65	0.23 (0.52)	48	0.14 (0.68)	0.1462

^a^ Change in median CFS at baseline and respective time point along with Wilcoxon signed rank test *P* value.

^b^ Number of patients with CFS data reported at baseline and at the relevant study visit.

**FIG. 2.**
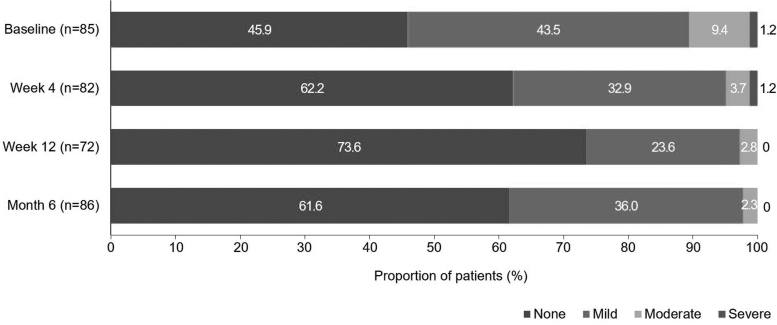
Change in severity of conjunctival hyperemia in the VISIONARY study population for Spain over time.

Conjunctival hyperemia severity was significantly reduced from baseline at week 4 (*P* = 0.0096), week 12 (*P* < 0.0001), and month 6 (*P* = 0.0037). The proportion of patients with no hyperemia increased from 45.9% at baseline to 61.6% at month 6 (Fig. 2). No patients had severe hyperemia at the end of the study period, and the proportion of patients with mild/moderate hyperemia was reduced from 52.9% at baseline to 38.8% at month 6.

Subjective symptoms were absent or of mild severity at baseline for >75% of patients concerning dry eye, irritation, and itching and for >90% for individuals when examining foreign body sensation and eye pain. At month 6, statistically significant reductions from baseline were reported concerning severity of dry eye symptoms (*P* = 0.0247), irritation (*P* = 0.0001), itching (*P* = 0.0082), foreign body sensation (*P* = 0.0202), and eye pain (*P* = 0.0403).

### Physician and patient assessments

Most investigators (79.3%) reported IOP control to be more effective with PF tafluprost/timolol FC treatment than with prior monotherapy at month 6. PF tafluprost/timolol FC treatment compliance at month 6 was perceived to be comparable with (69.6%) or greater than (29.3%) previous monotherapy. Patient-reported tolerability with PF tafluprost/timolol FC treatment was reported to be good/very good at week 4 (83.5%), week 12 (93.2%), and month 6 (91.0%).

### Safety outcomes

Overall, 6 AEs were reported by 6 patients during the 6-month study period, with 3 of these considered to be related to the study treatment: bradycardia, dizziness, and fatigue (Table 4). All reported AEs were nonserious, mild-to-moderate in severity, and had resolved or were resolving at the point of data cutoff, with the exception of 1 mild bradycardia case in which the outcome was unknown.

**Table 4.** Treatment-Related Adverse Events Reported During the Study Period

System/organ class	Number of treatment-related AEs
Neurological	
Dizziness	1
Cardiovascular	
Bradycardia	1
General disorders	
Fatigue	1

AEs, adverse events.

## Discussion

The current analysis of data from the VISIONARY study examined outcomes from 92 OAG and OHT patients in Spain and demonstrated statistically and clinically significant mean IOP reductions at month 6 and all interim study visits following initiation of PF tafluprost/timolol FC therapy. This 6-month, observational, multicenter prospective study demonstrated IOP-lowering efficacy with PF tafluprost/timolol FC treatment from week 4, regardless of the patient subgroup or prior monotherapy type used (PGA or beta-blocker), with clinical effectiveness, patient compliance, and patient-reported tolerability being rated highly with the study treatment.

Patients stepped up to the PF tafluprost/timolol FC from initial monotherapy with no washout period between treatments, which is reflective of clinical practice where treatment intensification is required to improve the management of OAG and OHT. All but 1 participant chose to instill their PF tafluprost/timolol FC treatment in the evening (1 drop, administered once per day), which may be indicative of the dosing recommendations provided by ophthalmologists to their patients in Spain.

The VISIONARY study was the first and largest European real-world study to specifically examine treatment outcomes among patients switching from PGA or beta-blocker monotherapy to an FC formulation in a stepwise manner, as recommended by the European Glaucoma Society.^12,34^ Other studies have examined the use of various topical FC glaucoma therapies in Spain, although it is not possible to compare outcomes from those investigations with the current analysis due to differences in trial design and study populations.^46–52^ In line with the outcomes reported for the full European VISIONARY population, month 6 reductions in mean IOP of >5 mmHg (22.3%), from baseline, were achieved among participants from Spain (*P* < 0.0001).^34^ Given the relatively moderate mean IOP at baseline among this cohort of patients (21.9 mmHg), the level of IOP-lowering efficacy achieved with PF tafluprost/timolol FC was considered remarkable compared with prior medication according to participating investigators. Prompt intervention to lower IOP, as well as the magnitude of IOP reduction achieved with treatment, are factors that have been identified as critical in the preservation of vision over an extended period in people with OAG.^53,54^ In both the Spanish and Europe-wide VISIONARY populations, the full IOP-lowering effect was evident at week 4 and maintained through week 12 and month 6, indicating that a meaningful IOP reduction may be achieved from the first month with PF tafluprost/timolol FC treatment, and these outcomes should be maintained for up to 6 months.^34^

Studies have demonstrated that a reduction in IOP of at least 11% is needed to achieve a change in visual field progression.^55^ Response rates in Spain showed that more than 60% of participants achieved IOP reductions ≥20%. Almost half of participants (47.8%) demonstrated that IOP reductions ≥25% and 37% reduced their IOP by 30% or more, showing that a high proportion of patients may achieve meaningful changes in IOP after switching to PF tafluprost/timolol FC from a PGA or beta-blocker monotherapy, with the associated benefits of potentially slowing disease progression and onset of irreversible sight loss.^53,54^

The mean baseline IOP in Spain was similar to that seen in the VISIONARY data published for cohorts in Hungary (20.8 mmHg) and the United Kingdom (22.0 mmHg).^40,41^ Given the real-world nature of the study, this provides an indication of local clinical practice and the pressures that would trigger ophthalmologists to conduct a switch to FC therapy in each of these countries.^40,41^ Cultural or lifestyle differences and local levels of compliance may also affect treatment outcomes with topical glaucoma therapies. The IOP reduction observed at month 6 in Spain (22.3%) was also similar to that seen in Hungary (22.6%), although slightly lower than that reported in the United Kingdom (24.9%).^40,41^ A much higher relative reduction in IOP from baseline was reported for patients in Russia (28.1%), which was likely due to ophthalmologists selecting patients with higher baseline IOP (23.8 mmHg) for the study.^42^

As also shown in the full VISIONARY study publication, participants diagnosed with POAG and OHT in Spain achieved significant reductions in IOP from baseline (*P* < 0.0001).^34^ However, the IOP reduction for patients in Spain with normal tension glaucoma and pseudoexfoliative could not be compared with the data shown in the full VISIONARY publication, due to the comparatively low patient numbers included in these diagnostic groups for the Spanish cohort.^34^

Most patients in Spain were treated with PGA monotherapy at baseline, and assessments according to prior treatment type showed that those patients demonstrated mean IOP reductions of 23.5% at month 6, which was comparable with outcomes from the European VISIONARY dataset for prior PGA users (23.6%).^34^ In contrast, patients in Spain who were previously treated with beta-blocker monotherapy demonstrated mean IOP reductions at month 6 of 14.6%, while those in the Europe-wide analysis achieved month 6 reductions of 28.5%. The variation in outcomes observed might be partly due to prior beta-blocker users in Spain having a lower baseline IOP (20.4 mmHg) compared with that of the full VISIONARY population (21.9 mmHg) as baseline pressure is predictive of the level of IOP reduction that may be demonstrated.^30,34^ However, patient numbers were relatively low in the prior beta-blocker group in Spain (*n* = 12) versus the full VISIONARY dataset (*n* = 161), so caution should be applied when comparing these data.^34^

As demonstrated in the Europe-wide analysis, most participants in Spain were enrolled in the study due to insufficient IOP control and/or progression of OAG and demonstrated significant IOP reductions at month 6 (*P* < 0.0001).^34^ Both the Europe-wide and Spanish VISIONARY analyses showed that significant IOP reductions were achieved with PF tafluprost/timolol FC treatment, regardless of whether patients reported dry eye symptoms at baseline (*P* < 0.0001), suggesting that the study treatment should be appropriate for patients with/without dry eye symptoms. However, analysis of data from Spain showed that a slightly higher relative reduction was observed among patients with baseline dry eye symptoms (23.1%) compared with those who did not have dry eye before starting PF tafluprost/timolol FC therapy (21.2%). Some studies have suggested that treatments that improve ocular surface health and reduce exposure to toxic agents, such as BAK, may provide enhanced IOP-lowering efficacy.^56–58^ Dry eye symptoms were significantly improved during the 6-month study period, and this may partly explain why patients experiencing dry eye at baseline showed slightly greater IOP reductions after a switch to a PF formulation. Improvements in tolerability might also have supported enhanced compliance and IOP-lowering outcomes. Although CFS was reduced from baseline at month 6, the change was not significant. This may have been because the Spanish VISIONARY population demonstrated a low mean baseline CFS score (0.3), which was below that of the full VISIONARY population (0.76), and there was little room for further reduction in severity.^34^ Significant improvements were observed concerning conjunctival hyperemia across the Spanish cohort at month 6 (*P* = 0.0037). Severity of conjunctival hyperemia was improved at each study visit, regardless of the prior PGA monotherapy used. Hyperemia levels appeared to be slightly lower at week 12 (when attendance at the clinic was optional) compared with month 6 when participants were mandated to attend the study visit. Conjunctival hyperemia data reported for month 6 may therefore be likely to be more representative of the full Spanish cohort, versus week 4 or 12 data. In addition, despite ocular symptoms being reported as generally mild or absent at baseline, significant improvements were shown concerning severity of dry eye (*P* = 0.0247), irritation (*P* = 0.0001), itching (*P* = 0.0082), foreign body sensation (*P* = 0.0202), and eye pain (*P* = 0.0403).

Significant increases were also seen regarding Schirmer's test and TBUT at month 6. These data further support the tolerability profile of the PF tafluprost/timolol FC at the ocular surface, as previously demonstrated in RCT and observational studies.^18,28,29,34–36^

At month 6, ophthalmologists considered IOP to be better controlled with the PF tafluprost/timolol FC medication than with the previous monotherapy in 79.3% and perceived compliance to the study medication to be better, versus prior monotherapy, in almost one-third of the patients. Compliance was scored as similar to the previous medication for 69.6% of the participants. The high levels of treatment compliance seen during the study may have been supported by patient perceptions of tolerability with PF tafluprost/timolol FC treatment, which were rated as good/very good in 91% of cases at month 6. In addition, just 3 treatment-related AEs were reported in Spain, all of which were mild-to-moderate in severity and none was serious. These data suggest that the treatment switch to PF tafluprost/timolol FC was well tolerated in the Spanish cohort. AEs were also mild-to-moderate in severity across the Europe-wide VISIONARY study population, and just 1 treatment-related AE was reported to be serious (status asthmaticus)—this event occurred in a patient from outside of Spain.^34^

Study limitations include the loss of information due to the observational nature of the research and participants choosing not to attend all visits, which is also an issue in routine practice. Previous studies have shown that IOP varies throughout a 24-h period, and data suggest that the greatest variations in IOP are seen outside of usual business hours.^59^ Unfortunately, it was not possible to examine the effects of PF tafluprost/timolol FC therapy on diurnal IOP variation during the study period as data were collected at routine ophthalmology appointments that were likely to have taken place within standard office hours. Again, this limitation reflects the challenges of glaucoma therapy and ongoing assessment of outcomes in clinical practice. This country-based analysis of the full VISIONARY dataset is also focused on a relatively small population, and larger scale studies of PF tafluprost/timolol FC treatment in Spain would be of benefit in further establishing the outcomes seen in the current study.

In conclusion, treatment outcomes with the PF tafluprost/timolol FC observed in ophthalmology clinics across Spain reflect those shown in the wider VISIONARY study population. Statistically and clinically significant IOP reductions were achieved in patients with OAG and OHT who were inadequately managed on a PGA or beta-blocker monotherapy, with treatment efficacy demonstrated from week 4 and sustained over 6 months. Over 60% of participants demonstrated an IOP reduction of more than 20%, which is well above the 11% reduction threshold that is believed to help slow progression of visual field deterioration.^55^ Most clinical signs and subjective symptoms were significantly improved from baseline. Safety data and patient-reported tolerability assessments showed PF tafluprost/timolol FC treatment to be generally well tolerated.
